# Supplementary material for: Safety of Early Bevacizumab Administration after Central Venous Port Placement for Patients with Colorectal Cancer
Source: Cancers (Basel). 2023 Apr 12;15(8):2264. doi: 10.3390/cancers15082264 (PMC10137085; doi:10.3390/cancers15082264)
Supplement: Supplementary file 1 [file cancers-15-02264-s001.zip › cancers-2281323-supplementary.pdf]

Supplementary Table S1. Univariate analysis for risk factors of CV port implantation-related complications.

Factor	Category	Univariate analysis		
		Odds ratio	95% CI	*P*
BEV initiation from implantation	< 7 days	1.00	Ref	Ref
	≤ 7 days	0.912	0.386–2.157	0.834
Age		1.003	0.964–1.044	0.865
Sex	Female	1.00	Ref	Ref
	Male	3.154	1.19–8.357	**0.021**
Insertion vein	Right internal jugular	1.00	Ref	Ref
	Right subclavian	2.67	0.301–23.69	0.378
	Left internal	0.271	0.014–5.387	0.392
Device type	PowerPort® ClearVUE® isp	1.00	Ref	Ref
	PowerPort® MRI	0.944	0.252–3.539	0.932
	Safe Guide™ Micro Needle Port	1.235	0.264–5.781	0.789
Procedure time	Procedure time	0.966	0.948–1.046	0.86
Performance status	0	1.00	Ref	Ref
	1	1.038	0.422–2.553	0.936
	2	0.597	0.071–4.992	0.634
Hypertension	Yes	1.647	0.630–4.309	0.309
Hyperlipidemia	Yes	1.935	0.499–7.508	0.340
Chronic kidney disease	Yes	0.325	0.016–6.724	0.467
Anticoagulant medication	Yes	1.491	0.508–4.376	0.467
Diabetes	Yes	1.286	0.345–4.791	0.708
Tumor location	Colon	1.00	Ref	Ref
	Rectum	0.856	0.345–2.121	0.737

Abbreviations: BEV, bevacizumab; CI, confidence interval.

Supplementary Table S2. Baseline patient characteristics in chemotherapy regimen with/without BEV groups.

Characteristics	Chemotherapy regimen with BEV (n = 184)	Chemotherapy regimen without BEV (n = 57)	*P*
Age, years	67 [32–85]	69 [23–85]	0.36
Sex; Female	96 (52)	20 (35)	0.09
Male	88 (48)	37 (65)	
Eastern Oncology Group Performance			
Status score	106 (58)	28 (49)	0.41
0	66 (36)	26 (46)	
1	12 (6)	3 (5)	
2			
Tumor site			
Colon	117 (64)	35 (61)	0.77
Rectum	67 (34)	22 (39)	
Hypertension			
Yes	39 (21)	18 (32)	0.11
No	145 (79)	39 (68)	
Hyperlipidemia			
Yes	14 (8)	5 (9)	0.78
No	170 (93)	52 (91)	
Chronic kidney disease			
Yes	9 (5)	3 (5)	0.91
No	175 (95)	54 (95)	
Diabetes			
Yes	19 (10)	11 (20)	0.06
No	165 (90)	45 (80)	
Anticoagulant medication			
Yes	29 (16)	10 (18)	0.75
No	155 (84)	47 (82)	
Body mass index	21.5 [16–42]	22.3 [16.2–30.7]	0.77

Supplementary Figure S1. Kaplan-Meier curve analysis for event-free survival for chemotherapy regimens with/without BEV.

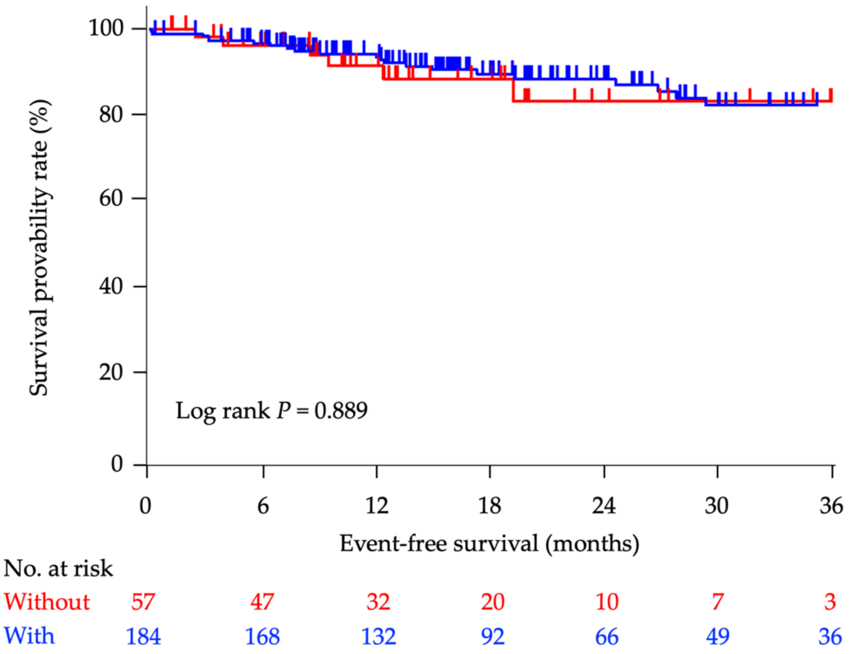

Kaplan-Meier analysis for event-free survival between the chemotherapy with BEV-containing regimen group (with) vs. that without BEV-containing regimen group (without), with no significant difference between the two (P = 0.889).

Abbreviations: BEV, bevacizumab.
